# A Practical Treatise on the Causes, Symptoms, and Treatment of Spermatorrhœa

**Published:** 1848-05

**Authors:** 


### BIBLIOGRAPHICAL NOTICES.

*A Practical Treatise on the Causes, Symptoms, and Treatment of Spermatorrhœa.* By M. Lallemand, formerly Professor of Clinical Surgery at the University of Montpellier; Member of the Royal Academy of Medicine of Paris, etc. etc. *Translated and Edited* by Henry I. McDougall, Member of the Royal College of Surgeons of England, etc. etc. 8vo. pp. 320. Lea & Blanchard. Philadelphia, 1848.

The subject to which the present volume is devoted, as far as we can discover, is little studied in this country; and the practice for the relief of the complaint, which, it appears to us, has been too generally resorted to by the author, is confined chiefly to cauterization. It is highly probable that with us the nasty habit of self-pollution is of far more rare occurrence than in older countries. The abundant means of occupation at the command of all, and the full remuneration of labour, which enable young people to enter into marriage at an early age, doubtless prevents this to a great extent. The masses, too, are better educated; and, in the constant incentives to enterprise peculiar to a new country and a teeming soil, they find enjoyments which prevent their lapsing into the solitary indulgences of less favoured communities. And yet, the vice exists; and the consequences, manifested in many serious derangements of the constitution, often present themselves to the notice of the physician, when it is probable the cause is not even suspected. The natural unwillingness of patients to disclose such weaknesses, especially when conscious of their origin, and the repugnance which most practitioners entertain towards such inquiries, particularly when the subjects, as is too generally the case, are both illiterate and filthy in their ordinary habits, sufficiently account for the general neglect of these investigations. Nevertheless, as we have observed, instances of the disease do occur, often unsuspected, and it is the duty of physicians to become acquainted with the symptoms which indicate it, the consequences of its neglect, that they may properly warn the unreflecting or ignorant sufferer, and the rational means of cure.

Although in these remarks we refer more particularly to cases arising from self-pollution, which is undoubtedly the most fruitful cause, the necessity for such knowledge is not the less where the disease arises from other influences.

The author's researches and observations on spermatorrhœa have long been before the profession, but in a foreign language, in detached essays, and, on other accounts, in a great degree inaccessible to American physicians. Mr. McDougall has conferred on the profession the benefit of a good English translation; and, in performing this task, he has judiciously omitted a large number of the cases which were not required for illustration, as well as much irrelevant matter. In this way, the useful contents of three large volumes are now comprised in a single octavo, of moderate size, and in a language understood by all our readers.

To those who would turn from the perusal of such a work, on ethical principles, or rather, we would say, from the erroneous notion that it is not the duty of the physician to lend his aid for the relief of ailments which result from moral perversion, we would observe, in the language of the author, that "diurnal (involuntary) pollutions are not always, as is generally believed, the result of venereal excesses, or of vicious habits. Many other varied causes, whose influence may be single, successive, or simultaneous, also give rise to them. Some of these cases are already understood, but of many others the medical world is completely ignorant; and these are the most dangerous, because their influence is the most difficult of appreciation." Although many of the cases narrated by the author have been omitted by the translator, sufficient are retained, on every point, to illustrate the principles set forth, and the effects of the practice pursued.

